# Promoter hypermethylation of SFRP1 as a prognostic and potentially predictive blood-based biomarker in patients with localized pancreatic ductal adenocarcinoma

**DOI:** 10.3389/fonc.2023.1211292

**Published:** 2023-06-02

**Authors:** Benjamin Emil Stubbe, Anders Christian Larsen, Poul Henning Madsen, Henrik Bygum Krarup, Inge Søkilde Pedersen, Søren Lundbye-Christensen, Carsten Palnæs Hansen, Jane Preuss Hasselby, Astrid Zedlitz Johansen, Ole Thorlacius-Ussing, Julia Sidenius Johansen, Stine Dam Henriksen

**Affiliations:** ^1^ Department of Gastrointestinal Surgery, Aalborg University Hospital, Aalborg, Denmark; ^2^ Department of Clinical Medicine, Aalborg University, Aalborg, Denmark; ^3^ Clinical Cancer Research Center, Aalborg University Hospital, Aalborg, Denmark; ^4^ Department of Molecular Diagnostics, Aalborg University Hospital, Aalborg, Denmark; ^5^ Unit of Clinical Biostatistics, Aalborg University Hospital, Aalborg, Denmark; ^6^ Department of Surgery, Copenhagen University Hospital - Rigshospitalet, Copenhagen, Denmark; ^7^ Department of Pathology, Copenhagen University Hospital – Rigshospitalet, Copenhagen, Denmark; ^8^ Department of Oncology, Copenhagen University Hospital – Herlev and Gentofte, Herlev, Denmark; ^9^ Department of Medicine, Copenhagen University Hospital – Herlev and Gentofte, Herlev, Denmark; ^10^ Department of Clinical Medicine, Faculty of Health and Medical Sciences, University of Copenhagen, Copenhagen, Denmark

**Keywords:** biomarker, pancreatic cancer, survival, epigenetic, DNA methylation, personalized therapy, blood-based, cfDNA

## Abstract

**Introduction:**

Current prognostic blood-based biomarkers for pancreatic adenocarcinoma (PDAC) are limited. Recently, promoter hypermethylation of SFRP1 (phSFRP1) has been linked to poor prognosis in patients with gemcitabine-treated stage IV PDAC. This study explores the effects of phSFRP1 in patients with lower stage PDAC.

**Methods:**

Based on a bisulfite treatment process, the promoter region of the SFRP1 gene was analyzed with methylation-specific PCR. Kaplan-Meier curves, log-rank tests, and generalized linear regression analysis were used to assess restricted mean survival time survival at 12 and 24 months.

**Results:**

The study included 211 patients with stage I-II PDAC. The median overall survival of patients with phSFRP1 was 13.1 months, compared to 19.6 months in patients with unmethylated SFRP1 (umSFRP1). In adjusted analysis, phSFRP1 was associated with a loss of 1.15 months (95%CI -2.11, -0.20) and 2.71 months (95%CI -2.71, -0.45) of life at 12 and 24 months, respectively. There was no significant effect of phSFRP1 on disease-free or progression-free survival. In stage I-II PDAC, patients with phSFRP1 have worse prognoses than patients with umSFRP1.

**Discussion:**

Results could indicate that the poor prognosis may be caused by reduced benefit from adjuvant chemotherapy. SFRP1 may help guide the clinician and be a possible target for epigenetically modifying drugs.

## 1 Introduction

Pancreatic cancer is expected to be the second leading cause of cancer-related death in the world, despite being only the 14^th^ most frequent cancer (1–3). Advances in both diagnostic approaches, surgical techniques, and intensified systemic adjuvant therapy have led to modest improvements in the 5-year survival rate from 8% in 2017 to 11% in 2022 (1, 4, 5). However, the bulk of the improvement is in patients with localized disease, where the 5-year survival has improved from 29% to 42% (1, 4). Unfortunately, only approximately 20% of patients with pancreatic cancer present with localized disease. The remaining 80% of patients are diagnosed with either locally advanced or metastatic disease, rendering curative treatment impossible (6).

The only curative treatment of pancreatic ductal adenocarcinoma (PDAC) is complete margin-negative resection of all tumor tissue (R0 resection) in combination with oncologic treatment. There is a high relapse rate within the first years of surgery despite a successful R0 resection (7–9). In a Danish cohort, patients curatively resected for PDAC between 2011 and 2016 had a median overall survival (OS) of 21.9 months (10). As such, even among curatively resected patients there is a poor prognosis compared to most other cancers (1). There is a lack of knowledge on the reasons behind this, and a need for biomarkers to guide clinicians toward treatment choices most suitable for the patient (6). The only routinely used diagnostic and prognostic biomarker in patients with PDAC is plasma levels of sialy-Lewis carbohydrate antigen 19-9 (CA19-9). Unfortunately, it is not cancer specific and 10% of the Caucasian population do not produce CA 19-9 (11, 12).

Secreted frizzled related protein-1 (SFRP1) is a tumor suppressor gene that mainly functions as an inhibitor of the oncogenic Wnt/ß-catenin pathway (13–15). The Wnt/ß-catenin pathway plays a crucial role in controlling cell proliferation and differentiation, and its dysregulation can cause the development of many diseases, including cancer (16). The absence of SFRP1 expression allows Wnt ligand to bind with the Fz receptor, leading to accumulation of ß-catenin and subsequent activation of downstream Wnt target genes (14, 15). The expression of SFRP1 is primarily regulated by promoter hypermethylation (17). As pancreatic carcinogenesis is heavily reliant on an activation of the Wnt/ß-catenin pathway, a promoter hypermethylation of SFRP1 (phSFRP1) is likely to occur early (18). This is supported by the observation of reduced SFRP1 expression in lower stage PDAC tumor tissue (19). Suppression of SFRP1 expression in cancer tissues is well established as a prognostic factor in several types of cancers, including breast, renal, biliary, head and neck, and PDAC (19–24). phSFRP1 has been proposed as an epigenetic biomarker for cancer detection, progression, and as an epigenetic treatment target (13, 25). However, there is sparse knowledge on the impact of phSFRP1 on survival as a blood-based analysis. Recently, we have reported that phSFRP1, measured in cell-free DNA (cfDNA), is a significant prognostic biomarker in patients with stage IV PDAC receiving palliative treatment with gemcitabine (24).

While most tumors are known to release circulating tumor DNA (ctDNA), metastatic tumors are associated with a substantially higher release compared to lower stage tumors (26, 27). It is currently unclear if phSFRP1, measured in cfDNA, impacts prognosis in lower stage PDAC (28, 29). Further, patients with stage I-II disease are potentially eligible for curative treatment, which may affect the impact of phSFRP1. In the present study we aimed to further explore the effects of phSFRP1 in patients with PDAC of stage I-II.

## 2 Materials and methods

### 2.1 Patients

This study was conducted in accordance with the REMARK (Reporting Recommendations for Tumor Marker Prognostic Studies) guidelines (30). Retrospectively, a cohort was defined comprising patients with histologically verified stage I or II PDAC, treated with either FOLFIRINOX, gemcitabine, or best supportive care (BSC). Serum or EDTA plasma samples were received from two Danish biobanks and subsequently analyzed. The GIVTE study (“Venous Thromboembolism and Haemostatic Disturbances in Patients with Upper Gastrointestinal Cancer”; ClinicalTrials.gov ID NCT00660205) and the BIOPAC study (“BIOmarkers in patients with PAncreatic Cancer (BIOPAC) – can they provide new information of the disease and improve diagnosis and prognosis of the patients”; ClinicalTrials.gov ID NCT03311776; www.herlevhospital.dk/BIOPAC/).

The GIVTE study was a Danish study examining the prevalence of venous thromboembolism at diagnosis of various upper gastrointestinal cancers, including pancreatic cancer. Patients were included consecutively upon diagnosis and before treatment with either surgery or chemotherapy at the Department of Surgery, Aalborg University Hospital between February 2008 and February 2011. The GIVTE study protocol is approved by the Danish Ethics Committee (VEK, j.nr. N-20080002).

The BIOPAC study is a prospective Danish multicenter open cohort study with ongoing enrollment of patients who present with pancreatic cancer. Patients in the present study were included at the time of their PDAC diagnosis, before treatment with surgery or chemotherapy between September 2011 and February 2016 at the Department of Surgery, Rigshospitalet or Department of Oncology, Copenhagen University Hospital – Herlev and Gentofte. The BIOPAC study protocol is approved by the Danish Ethics Committee (VEK, j.nr. KA-20060113) and the Danish Data Protection Agency (j.nr. 2012-58-0004; HGH-2015-027; I-Suite j.nr. 03960; and PACTICUS P-2020-834). The study was conducted in accordance with the Declaration of Helsinki. Clinical data was not received until methylation analysis was completed.

### 2.2 Methylation analysis

In the GIVTE study, EDTA plasma was obtained after centrifugation (4.000 rpm, 20 min, 4°C). In the BIOPAC study, serum was obtained after centrifugation (2.300 G, 10 min, 4°C). Blood samples were centrifuged and frozen at -80°C within two hours of sampling time. All methylation analyses of samples from both cohorts were performed by a single expert laboratory scientist at the Department of Molecular Diagnostics, Aalborg University Hospital, Denmark.

Samples were treated with a modified bisulfite treatment protocol developed by our group, as previously described (24, 31). Extraction, deamination of cell-free (cf)DNA, and two rounds of PCR amplification were performed. The hemimethylated MEST transcript variant 1 was used as a reference gene. An initial PCR amplification was performed to expand deaminated DNA using the outer methylation-specific primer for SFRP1, **Supplementary Table 1**. This was followed by a series of individual PCR reactions using the inner methylation-specific primers and probes. SFRP1 was analyzed with a panel of other genes (32).

Following PCR amplification, data on SFRP1 promoter methylation status was dichotomized. A promoter hypermethylated gene was defined as a sample with any detectable cycle threshold value within 45 cycles. A promoter unmethylated gene was defined as a sample with an undetectable cycle threshold value. This dichotomization has previously been demonstrated not to lead to significant loss of information (32).

### 2.3 Statistical methods

Patients of stage I-II were pooled and stratified into subgroups according to SFRP1 methylation status and resection status. A R0 resection was defined as a resection with no macroscopic remains in the primary tumor bed and a microscopically tumor-negative margin of at least 1.5 mm. A R1 resection was defined as the removal of all macroscopic tumor tissue, but with microscopic margins positive for residual tumor. Unresected was defined as patients who were either inoperable due to comorbidity or where disease was initially judged as resectable but found unresectable upon explorative laparotomy.

Missing data regarding ECOG PS was imputed by Multiple Imputation by Chained Equations with Predictive Mean Matching, using age as a predictor with 100 imputations (33).

Kruskal-Wallis tests and Pearson Chi-Squared tests were used to compare continuous and categorical variables, respectively.

OS was calculated from the time of pretreatment blood sampling until death of any cause or end of follow-up on June 14, 2022. Disease-free survival (DFS) (in R0-resected patients) was calculated from the date of successful R0 resection until disease recurrence, death of any cause, or end of follow-up. Progression-free survival (PFS) (R1 or unresected patients) was calculated from the time of pretreatment blood sampling until progression of disease, death of any cause, or end of follow-up on June 14, 2022. Patients were followed according to Danish guidelines and received CT-scans only on suspicion of recurrence.

The primary outcome, survival time, was analyzed with established methods for survival analysis. As the proportional hazard assumption was violated, comparisons were quantified using Restricted Mean Survival Time (RMTS) (34). The RMST is a measure of the average survival from time 0 to a particular time point. Twelve- and 24-month RMTS were chosen to reflect the mOS of this patient group (10). The RMST was calculated and analyzed using the pseudo-observation method (35). Crude and adjusted regression analyses based on generalized linear models were performed using the pseudo-observation method. Standard error, p-values and confidence intervals were calculated using robust variance estimation.

Crude models were performed for dichotomized SFRP1 methylation status and the covariates age > 65 years, Eastern Cooperative Oncology Group (ECOG) performance status above 1 (PS), sex (female or male), treatment with adjuvant chemotherapy (no or yes), stage of disease (I or II), CA 19-9 (below or above the median), and resection status (R0 resection, R1 resection, or unresected patients). Following the crude analysis, we performed an adjusted analysis with the same covariates.

Kaplan-Meier survival curves with log-rank tests were used to graphically illustrate survival.

A *p*-value < 0.05 was considered statistically significant, and 95% confidence intervals (CI) were employed for all tests where applicable. All statistical calculations were carried out in either Stata v. 16, StataCorp, LLC, TA, USA or R version 4.2.2: A language and environment for statistical computing. R Foundation for Statistical Computing, Vienna, Austria.

## 3 Results

### 3.1 Patient characteristics

A total of 211 patients (BIOPAC (*n* = 171) and GIVTE (*n* = 40) with stage I-II PDAC were included in the study. Patients were stratified by resection status and SFRP1 promoter hypermethylation status. Information regarding ECOG PS was missing in 28 patients, all patients with missing PS had pancreatic resection. There was a higher proportion of patients with stage I disease in the R1-resection and unresected strata (*p* = 0.04). Less patients in the R1-resected and unresected strata received chemotherapy (*p* = 0.02). No significant differences between the groups according to SFRP1 promoter hypermethylation status were found in age, sex, PS, weight, BMI, CA19-9, or location of the primary tumor (**Table 1**). There was no significant difference in the frequency of SFRP1 promoter hypermethylation according to resection status (*p* = 0.27). Ten patients were below 50 years of age. One patient below 50 years of age had phSFRP1 and died 8 months after their R0-resection.

**Table 1:** Characteristics of patients with PDAC according to resection status and SFRP1 promoter methylation status.

Characteristics	R0-resected		R1-resected		Unresected		All	*p*-value
	umSFRP1	phSFRP1	umSFRP1	phSFRP1	umSFRP1	phSFRP1		
	(n = 125)	(n = 30)	(n = 23)	(n = 4)	(n = 20)	(n = 9)	(n = 211)	
**Age, years (mean, range)**	66 (37-84)	67 (38-81)	65 (48-81)	69 (61-79)	68 (55-84)	72 (55-82)	67 (37-84)	0.36^a^
Sex								0.73^b^
Male	53 (42%)	12 (40%)	10 (43%)	2 (50%)	7 (35%)	6 (67%)	90 (43%)	
Female	72 (58%)	18 (60%)	13 (57%)	2 (50%)	13 (65%)	3 (33%)	121 (57%)	
**Weight, (mean, range)^1^**	71 (43-119)	72 (51-105)	77 (57-110)	75 (50-92)	65 (47-82)	69 (58-80)	71 (43-119)	0.42^a^
BMI^2^								0.10^b^
< 18.5	5 (4%)	2 (7%)	0 (0%)	0 (0%)	4 (20%)	0 (0%)	11 (5%)	
18.5-25	67 (57%)	19 (66%)	15 (68%)	1 (25%)	10 (50%)	3 (33%)	115 (57%)	
> 25	45 (38%)	8 (28%)	7 (32%)	3 (75%)	4 (20%)	2 (22%)	69 (34%)	
**CA 19-9, U/ml, (median, range)**	176 (1-45500)	168 (6-7830)	284 (3-3590)	27 (4-162)	158 (3-5930)	28 (3-966)	167 (3-45500)	0.33^a^
**Stage I PDAC**	9 (7%)	4 (13%)	0 (0%)	1 (25%)	5 (25%)	2 (22%)	21 (10%)	0.04^b^
**Stage II PDAC**	116 (93%)	26 (87%)	23 (100%)	3 (75%)	15 (75%)	7 (78%)	190 (90%)	
Type of chemotherapy								0.02^b^
No chemotherapy	18 (14%)	5 (17%)	10 (43%)	3 (75%)	2 (10%)	3 (33%)	41 (19%)	
Gemcitabine	103 (82%)	24 (80%)	13 (57%)	1 (25%)	17 (85%)	6 (67%)	164 (78%)	
FOLFIRINOX	4 (3%)	0 (0%)	0 (0%)	0 (0%)	1 (5%)	0 (0%)	5 (2%)	
Unknown	0 (0%)	1 (3%)	0 (0%)	0 (0%)	0 (0%)	0 (0%)	1 (0%)	
**Series of chemotherapy (mean, range)^3^**	5 (0-22)	5 (0-15)	4 (0-9)	2 (0-9)	6 (0-14)	5 (0-21)	5 (0-22)	0.20^a^
Location of primary tumor								0.85^b^
Caput	102 (82%)	26 (87%)	20 (87%)	4 (100%)	17 (85%)	4 (44%)	173 (82%)	
Corpus	7 (6%)	1 (3%)	2 (9%)	0 (0%)	2 (10%)	0 (0%)	12 (6%)	
Cauda	7 (6%)	2 (7%)	1 (4%)	0 (0%)	1 (5%)	2 (22%)	13 (6%)	
Diffuse	7 (6%)	1 (3%)	0 (0%)	0 (0%)	0 (0%)	0 (0%)	8 (4%)	
Papilla	1 (1%)	0 (0%)	0 (0%)	0 (0%)	0 (0%)	0 (0%)	1 (0%)	
Unknown	1 (1%)	0 (0%)	0 (0%)	0 (0%)	0 (0%)	3 (33%)	4 (2%)	
ECOG Performance Status								0.19^b^
0	66 (53%)	12 (40%)	8 (35%)	3 (75%)	11 (55%)	4 (44%)	104 (49%)	
1	33 (26%)	12 (40%)	9 (39%)	1 (25%)	5 (25%)	2 (22%)	62 (29%)	
2	6 (5%)	1 (3%)	1 (4%)	0 (0%)	4 (20%)	3 (33%)	15 (7%)	
3	2 (2%)	0 (0%)	0 (0%)	0 (0%)	0 (0%)	0 (0%)	2 (1%)	
Unknown	18 (14%)	5 (17%)	5 (22%)	0 (0%)	0 (0%)	0 (0%)	28 (13%)	

phSFRP1, patients with SFRP1 promoter hypermethylation; umSFRP1, patients without SFRP1 promoter hypermethylation. a Kruskal–Wallis one-way analysis of variance. b Pearson chi-square test. ^1^Missing in 22 patients, ^2^Missing in 16 patients, ^3^Missing in 5 patients.

### 3.2 Overall survival

**Figure 1:**
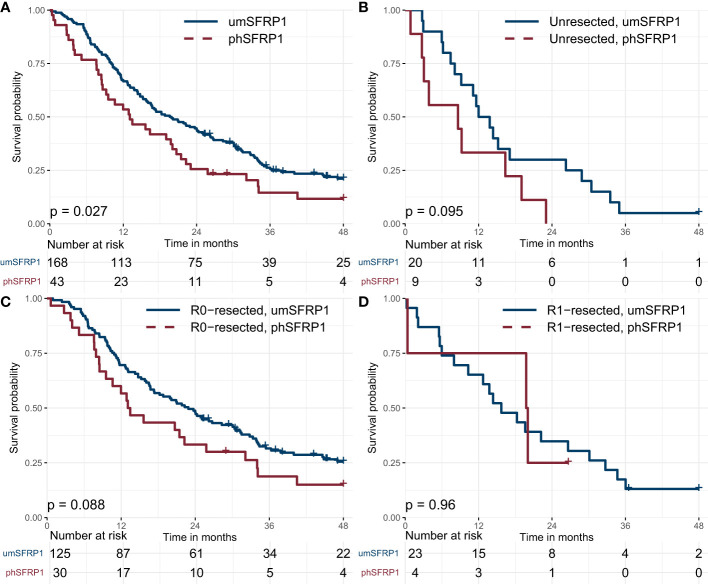
Kaplan-Meier survival curves for patients with stage I-II PDAC, grouped by SFRP1 promoter hypermethylation status. **(A)** Association between phSFRP1 and survival among all patients with stage I-II PDAC. **(B)** Association between phSFRP1 and survival among patients with unresected stage I-II PDAC. **(C)** Association between phSFRP1 and survival among patients with R0-resected stage I-II PDAC. **(D)** Association between phSFRP1 and survival among patients with R1-resected stage I-II PDAC. phSFRP1, patients with SFRP1 promoter hypermethylation; umSFRP1, patients without SFRP1 promoter hypermethylation. Risk table shows the number of patients at risk in 6-month intervals.

During the follow-up period, 195 (92%) patients had died. In the entire cohort, the 12- and 24-month survival was 64% and 41%, respectively, with a median (m)OS of 18.2 months. R0-resected patients had a mOS of 21.0 months compared to 13.8 months in R1-resected patients and 11.6 months in unresected patients. phSFRP1 patients had a mOS of 13.1 months compared to 19.6 months in unmethylated SFRP1 (umSFRP1) patients, **Figure 1A**.

The mOS was shorter in unresected patients with phSFRP1 compared to umSFRP1 (8.6 months vs. 12.0 months), **Figure 1B**. R0-resected patients with phSFRP1 had a shorter mOS of 13.1 months compared to 22.9 months in patients with umSFRP1, **Figure 1C**. However, R1- resected patients with phSFRP1 had a longer mOS compared to patients with umSFRP1 (19.8 months vs. 15.7 months), **Figure 1D**. The mOS of R0-resected patients with umSFRP1 was 10.9 months longer than in unresected patients. In contrast, the mOS of R0- resected patients with phSFRP1 was only 4.5 months longer than in unresected patients.

Kaplan-Meier survival curves according to resection status, stage, ECOG PS, Age, CA 19-9 and treatment can be found in **Supplementary Figures 1A–F**.

**Figure 2:**
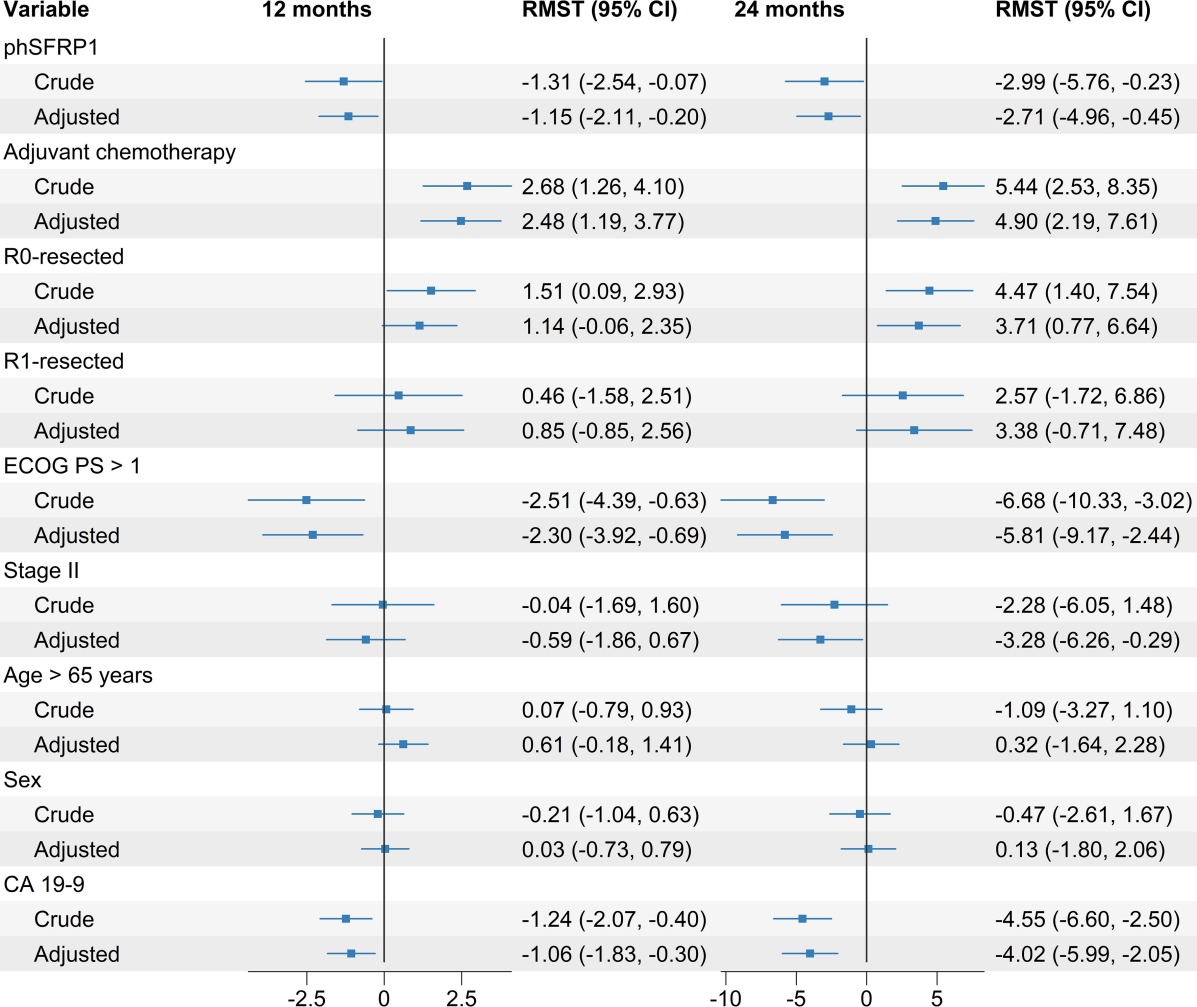
Crude and adjusted differences in Restricted Mean Survival Time (RMST) in months between groups according to SFRP1 methylation status. Adjusted regression models included SFRP1 methylation status as well as the prognostic factors age > 65 years, PS, sex, treatment with adjuvant chemotherapy, stage of disease, CA 19-9 above the median, and resection status. Differences are calculated from baseline up to 12 and 24 months, respectively. Increased survival is indicated by positive number in months, decreased survival is indicated by negative numbers.

In crude regression models, phSFRP1 was significantly associated with loss of 1.31 months (95% CI -2.54, -0.07) and 2.99 months (95% CI -5.76, -0.23) of life within the first 12 and 24 months, respectively (**Figure 2**). R0-resection status, treatment with adjuvant chemotherapy, PS > 1 and CA 19-9 > 167 were significantly associated with survival at both time points. Disease stage, age above 65, sex, and R1-resection status were not significantly associated with survival.

Following the crude regression models, we performed adjusted regression models, adjusting for the effects of other prognostic factors. The variables included were age > 65 years, PS, sex, treatment with adjuvant chemotherapy, stage of disease, CA 19-9 above the median, and resection status.

In the adjusted regression models phSFRP1 was significantly associated with a loss of 1.15 months (95% CI -2.11, -0.20) and 2.71 months (-4.96, -0.45) of life at 12 and 24 months, respectively. A PS > 1, CA 19-9 value > 167, and treatment with adjuvant chemotherapy were also significantly associated with survival at both time points. R0-resection status and stage were significantly associated with longer survival at 24 months, but not at 12 months. R1-resection, age > 65 years, and sex were not associated with survival at either time point (**Figure 2**). An adjusted model using only cases with complete information on ECOG PS did not change results substantially, see **Supplementary Figure 2**.

### 3.3 Disease-free survival

**Figure 3:**
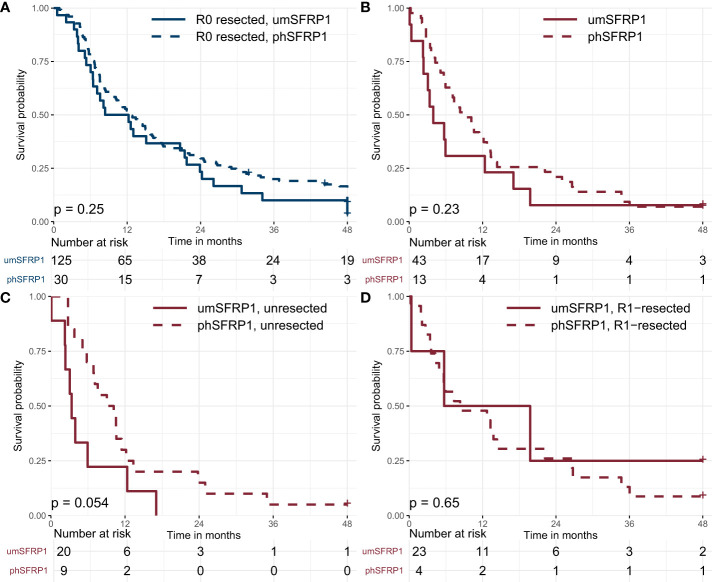
Kaplan-Meier curves showing disease-free survival and progression-free survival in stage I-II PDAC, stratified by SFRP1 promoter hypermethylation status. **(A)** Association between phSFRP1 and selected prognostic factors on Disease-free survival in R0-resected patients. **(B)** Association between phSFRP1 and selected prognostic factors on Progression-free survival in R1 and unresected patients. **(C)** Association between phSFRP1 and selected prognostic factors on Progression-free survival in unresected patients. **(D)** Association between phSFRP1 and selected prognostic factors on Progression-free survival in R1-resected patients. Risk table shows the number of patients at risk in 6-month intervals. phSFRP1, patients with SFRP1 promoter hypermethylation; umSFRP1, patients without SFRP1 promoter hypermethylation.

The 12- and 24-month DFS was 52% and 29%, respectively, with a median DFS of 12.6 months. The median PFS was 12.2 months in patients with phSFRP1, compared to 12.9 months in patients with umSFRP1. Kaplan-Meier curves are shown in **Figure 3A**.

**Figure 4:**
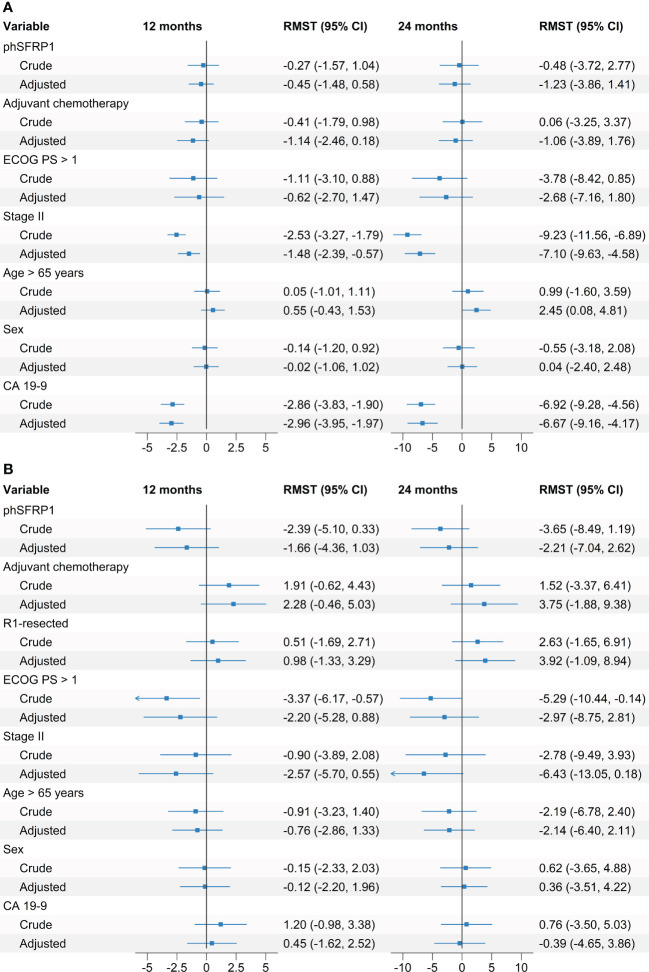
Crude and adjusted differences in Restricted Mean Disease-Free Survival and Restricted Mean Progression-Free survival according to SFRP1 methylation status. Adjusted regression models included SFRP1 methylation status as well as the prognostic factors age > 65 years, PS, sex, treatment with adjuvant chemotherapy, stage of disease, CA 19-9 above the median, and resection status. **(A)** Association between phSFRP1 and selected prognostic factors on Disease-Free survival. **(B)** Association between phSFRP1 and selected prognostic factors on Progression-Free survival. Differences between groups are calculated in months from baseline up to 12 and 24 months. Increased survival is indicated by positive number in months, decreased survival is indicated by negative numbers.

In a crude model, phSFRP1 was not significantly associated with loss of DFS neither at 12 months (RMST -0.27 months, 95% CI -1.57, 1.04) nor at 24-months (RMST -0.48 months, 95% CI -3.72, 2.77). Stage of disease and CA 19-9 > 167 were significantly associated with reduced DFS at both time points. Neither treatment with chemotherapy, ECOG PS > 1, age > 65 or sex were significantly associated with loss of DFS (**Figure 4A**).

In the adjusted models, phSFRP1 was not significantly associated with a loss of DFS neither at 12 months (RMST -0.45 months, 95% CI -1.48, 0.58) nor at 24-months (RMST -1.23 months, 95% CI -3.86, 1.41). Stage and CA 19-9 > 167 were significantly associated with reduced DFS at both 12 and 24 months. Age > 65 was significantly associated with an increased DFS of 2.45 months (95% CI, 0.08, 4.81) at the 24-month point, but not at the 12-month point (RMST 0.55, 95% CI -0.43, 1.53). Neither treatment with chemotherapy, ECOG PS > 1 nor sex were significantly associated with shorter DFS.

### 3.4 Progression-free survival

The 12- and 24-month PFS was 38% and 18%, with a median PFS of 7.3 months. The median PFS was 3.9 months in patients with phSFRP1 compared to 9.0 months in patients with umSFRP1, **Figure 3B**. The median PFS was 3.4 months in unresected patients with phSFRP1 compared to 9.0 months in patients with umSFRP1, **Figure 3C**. The median PFS was 5.7 months in R1-resected patients with phSFRP1, compared to 8.3 months in patients with umSFRP1, **Figure 3D**.

In the crude analysis, phSFRP1 was not significantly associated with short PFS at neither 12 months (RMST -2.39 months, 95% CI -5.1, 0.33) nor at 24 months (RMST -3.65 months, 95% CI: -8.49, 1.19). A ECOG PS > 1 was significantly associated with a loss of 3.37 (95% CI -5.10, 0.33) and 5.29 (95% CI -8.49, 1.19) months of PFS at 12 and 24 months, respectively. Neither resection status, disease stage, chemotherapy, age > 65, CA 19-9 > 167 nor sex were significantly associated with shorter PFS at either 12 or 24 months (**Figure 4B**).

In the adjusted model, phSFRP1 was not significantly associated with short PFS at neither at 12 months (RMST = -2.21 months, 95% CI -7.04, 2.62) nor at 24 months (RMST = -1.66 months, 95% CI -4.36, 1.03). Neither EGOC PS > 1, resection status, disease stage, treatment with chemotherapy, CA 19-9 > 167, age or sex were significantly associated with shorter PFS at either 12 or 24 months.

## 4 Discussion

In the present study we examined the association of phSFRP1 with survival in patients with stage I-II PDAC. Our results showed that phSFRP1 has a significant negative impact on survival in these patients. This supports our previous findings in patients with stage IV PDAC, and suggests that phSFRP1 may also be a clinically relevant biomarker in patients with stage I and II PDAC (24).

We expected a successful R0 resection to be the most important prognostic factor for the patients’ long-term survival. Therefore, patients with stages I-II were pooled, as both are offered curative resection if possible, and subsequently stratified by their resection status. Not surprisingly, our results showed that patients who received a successful R0 resection lived longer than patients who did not. The mOS in our cohort is similar to the mOS in all patients with stage I-II PDAC in Denmark in the same time period, suggesting the generalizability of our findings to at least the Danish population (10).

In both crude and adjusted models, we found phSFRP1 to be significantly associated with shorter survival in patients with stage I-II.

R0-resected patients with umSFRP1 had the longest mOS, while unresected patients with phSFRP1 had the shortest. In R1-resected patients, those with phSFRP1 had a mOS longer than umSFRP1 patients, but this should be interpreted cautiously as the exposure group was very small.

An R0 resection was associated with a larger survival benefit in patients with umSFRP1, compared to patients with phSFRP1. R0-resected patients with phSFRP1 had a shorter mOS than R1-resected patients with umSFRP1, and only slightly better than unresected patients with umSFRP1. The 12- and 24-month survival of R0-resected patients with phSFRP1 (57% and 33%) was approximately equal to that seen in unresected patients with umSFRP1 (50% and 30%). This suggests that phSFRP1 status could potentially rival an R0 resection as a prognostic marker in patients with stage I-II PDAC. It could also indicate that patients with phSFRP1 may benefit less from surgery than patients with umSFRP1. If this is the case, it raises the question of whether surgical intervention is always beneficial for these patients when weighed against possible complications and long-term adverse events. Pancreatoduodenectomy (Whipple’s procedure) is a major abdominal procedure, with a substantial risk of major complications (36). A decrease in quality of life in both physical and psychosocial functioning can be expected in at least the first 3 months following pancreatoduodenectomy, eventually recovering to baseline after 3-6 months (36). Both the risks of surgery and postoperative quality of life for the patients should be considered carefully, especially if there is only a relatively minor survival benefit.

However, this raises the question of the underlying reason for the poor prognosis in R0-resected patients with phSFRP1. The effect of phSFRP1 in R0-resected patients could indicate the influence of other mechanisms than simply an aggressive tumor subtype, as all tumor material was resected with negative margins. Intuitively, one explanation for this phenomenon could be that phSFRP1 is a proxy of micro metastases, leading to early recurrence and death. However, we found no significant differences in DFS according to SFRP1 methylation status at neither 12 nor 24 months. With a point estimate close to 0 and confidence intervals distributed evenly around 0, this indicates no clinically relevant effect on DFS. The majority of patients who experience recurrence do so within two years after resection (9). As such, based on our results, it is unlikely that SFRP1 methylation status substantially impacts disease recurrence after resection.

Recurrence is most likely caused by micro metastases undetectable at diagnosis. This theory has led to intensified adjuvant treatment regimens following resection and fairly substantial improvements in survival (1, 4, 5). As phSFRP1 was not linked to shorter DFS in this cohort, the shorter survival of patients with phSFRP1 is unlikely to be linked to micro metastases.

A trend towards shorter PFS was seen in patients with phSFRP1. The small groups are likely the cause of the insignificant differences despite the large effect sizes. While not significant, the left-shifted confidence intervals could indicate a clinically relevant negative effect of phSFRP1 on PFS.

A more likely explanation for the shorter OS in R0-resected patients with phSFRP1 could be a reduced response to subsequent adjuvant chemotherapy in the patients who experience recurrence. A potential mechanism of action of phSFRP1 is through an upregulation of the oncogenic Wnt/ß-catenin pathway (17). SFRP1 modulates the Wnt signalling pathway through several mechanisms (14, 15, 37, 38). A reduction in SFRP1 expression promotes the binding of Wnt ligand to the Fz receptor (15). This binding causes the phosphorylation of the lipoprotein receptor-related protein, leading to recruitment and activation of Dishevelled proteins (39). Activated Dishevelled polymers inactivate the destruction complex, leading to accumulation of ß-catenin and activation and transcription of Wnt target genes (39). Previous literature has linked an activation of the Wnt/ß-catenin pathway to chemoresistance (40, 41). Additionally, phSFRP1 has been linked to chemotherapy resistance (24, 42, 43).

Theoretically, this could prove a potential treatment target, as reactivation has been linked to resensitization (42). Potential treatment modality is the use of hypomethylating drugs, such as decitabine and azacytidine, which have become widely used in cases of acute myeloid leukemia and higher-risk myelodysplastic syndrome that are not eligible for intense chemotherapy or allogeneic hematopoietic stem cell transplantation (44). The drugs work by different mechanisms, but both functions to re-express silent tumor suppressor genes and have proven superior to conventional treatment regimens in these patients, improving survival and possibly inducing complete remission (44). Such hypomethylating agents could potentially also provide value in solid tumors with promoter hypermethylation-mediated silencing of tumor suppressor genes. This has been previously suggested as a possibility in patients with non-small cell lung cancer (45). Several clinical trials are currently ongoing examining epigenetically modifying drugs in PDAC (46).

A problem, however, with these treatments are their shotgun approach, which may also activate latent oncogenes by demethylation (47). Additionally, a recent study in lymphoma cell lines demonstrated some promoter regions to remain hypermethylated despite treatment with hypomethylating agents (48). This could suggest that the promoter hypermethylated silencing is actively maintained by cancer cells, which would provide further challenges in the implementation of hypomethylating treatments.

Another approach is the recently proposed concept of tumor mimetics for class 2 tumor suppressor genes (49). The approach seeks to phenotypically mimic the action of secreted proteins, such a SFRP1. A novel mimetic compound has been identified which successfully limited growth of cancer cells with promoter hypermethylation-mediated SFRP1 downregulation (49).

A recent paper described a detailed molecular analysis of crucial regions of SFRP1 CpG site hypermethylation with identification of a core CpG island (CGI2) of particular interest (50). The investigators were able to show a strong inverse correlation between DNA methylation status of the CGI2 and SFRP1 mRNA expression both in silico and *in vitro*, providing stronger evidence for the regulatory mechanism of methylation in PDAC. They found low DNA methylation of the CGI2 to favor overall survival in silico and proposed that a pyrosequencing assay could replace cfDNA analysis to investigate phSFRP1 and predict chemoresistance in PDAC. This is likely feasible in most resected patients, and tissue analysis provides several benefits compared to cfDNA. However, at least in Denmark, resected patients remain a minority of cases – approximately 20% (10). This leaves a continued need for a more minimally invasive approach in most cases where either the tumor is unresectable, the tumor tissue retrieved is insufficient, or the patient is unfit for surgery.

Liquid biopsies are certainly not without their limitations, being inherently reliant on the tumor to release sufficient ctDNA to be technically detectable. This challenge is enhanced in patients with lower stages of disease, as they are known to release less ctDNA (26, 27). However, here we showed that even in stage I-II PDAC, phSFRP1 is both measurable in cfDNA and indicates a significantly poorer prognosis.

Future clinical studies are required to evaluate the exact mechanisms of phSFRP1 in patients with PDAC, and whether the biomarker is a surrogate for poor efficacy of chemotherapy or a more aggressive tumor phenotype. Studies are planned to examine the effects of phSFRP1 in larger cohorts of patients and transfer the analysis to a fully quantitative ddPCR-based approach, which could improve both sensitivity and specificity. Additionally, this would allow for more comprehensive validation of the prognostic impact of the level of methylation.

This was a retrospective study, which could cause selection bias. This risk is limited by the prospective collection of data in the original studies and the blinded methylation analysis. Further, there is limited censoring, as most patients were followed until death (93%). Of remaining patients, the shortest follow-up was 25 months. CA 19-9 levels were dichotomized according to the median, leading to easier interpretation at the expense of some loss of information. Notably, most of this patient cohort was treated with curative intentions, the success of which considerably impacts survival. PS was not registered for 28 patients. However, all patients with missing PS underwent surgery, with 23 receiving R0-resections and thus likely being in a good PS. Data regarding DFS and PFS are limited by the guidelines in the Danish follow-up program, as CT-scans are only performed upon signs of recurrence. It is possible that regular screening with CT-scans could impact DFS and PFS to some degree.

## 5 Conclusion

In conclusion, our results emphasize the importance and relevance of promoter hypermethylation of SFRP1 in cfDNA in the outcomes of patients with stage I-II PDAC. The presence of phSFRP1 might be associated with some of the mechanisms influencing prognosis in patients curatively resected for PDAC. The results indicate that patients with phSFRP1 may benefit less from adjuvant chemotherapy, compared to patients with umSFRP1. However, these findings require validation in larger, preferably prospective cohorts. If confirmed, SFRP1 could be a potential epigenetic treatment target, and its methylation status could potentially facilitate personalized treatment of patients with stage I-II PDAC.

## Data availability statement

The raw data supporting the conclusions of this article will be made available by the authors, without undue reservation.

## Ethics statement

The studies involving human participants were reviewed and approved by Regional Research Ethics Committee of Northern Denmark (approval number: N-20130037). The patients/participants provided their written informed consent to participate in this study.

## Author contributions

BS: writing - original draft, visualization, formal analysis, software; AL: writing - review and editing, investigation, resources; PM: investigation, resources, methodology. HK: writing - review and editing, methodology, resources; IP: writing - review and editing, methodology, resources; SL-C: formal analysis, visualization, writing – review and editing; CH: writing - review and editing, resources. JH: writing - review and editing, resources. AJ: writing - review and editing, resources. OT-U: supervision, conceptualization, writing - review and editing, methodology. JJ: writing - review and editing, resources. SH: supervision, writing - review and editing, methodology, investigation, resources. All authors contributed to the article and approved the submitted version.
