# Supplementary material for: Promoter hypermethylation of SFRP1 as a prognostic and potentially predictive blood-based biomarker in patients with localized pancreatic ductal adenocarcinoma
Source: Front Oncol. 2023 Jun 2;13:1211292. doi: 10.3389/fonc.2023.1211292 (PMC10272559; doi:10.3389/fonc.2023.1211292)
Supplement: Supplementary file 1 [file DataSheet_1.docx]

**Supplementary Table 1.** DNA sequences and positions for primers and probes.

| Primers and probe | Size of amplicon | Positions | DNA Sequence |  |
| --- | --- | --- | --- | --- |
| SFRP1 Am |  | 41309567-41309584 | GAG GCG ATT GGT TTT CGC |  |
| SFRP1 Bm | 149 | 41309435-41309450 | CGC GAC ACT AAC TCC G |  |
| SFRP1 M beacon |  | 41309482-41309493 | (HEX)CGC GAT G+GT T+CG +GTC G+TA ATC GCG(Dabcyl) |  |
| SFRP1 M1 |  | 41309508-41309525 | GGA GTT GAT TGG TTG CGC |  |
| SFRP1 M2 | 90 | 41309435-41309450 | CGC GAC ACT AAC TCC G |  |
| +; Locked nucleic acid Am; First round outer methylation specific forward primer for the nested/semi‐nested PCR  Bm; First round outer methylation specific reverse primer for the nested/semi‐nested PCR  M1; Inner methylation specific forward primer for the array  M2; Inner methylation specific reverse primer for the array  M beacon; methylation specific probe | | | | |


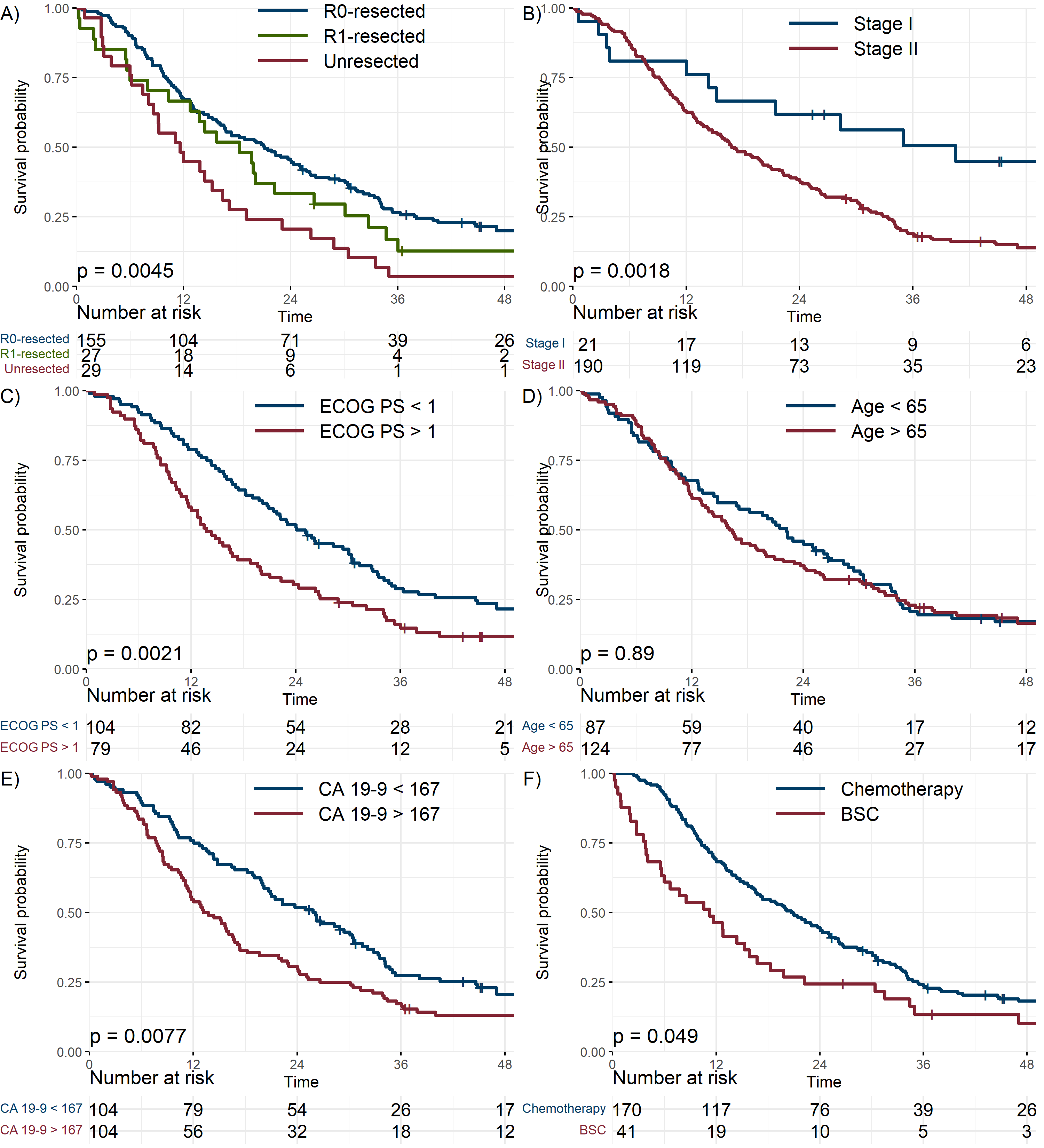


**Supplementary Figure 1.** Kaplan-Meier survival curves for patients with stage I-II PDAC, grouped according to known prognostic factors. A) Resection status. B) Stage. C) ECOG Performance Status below or above 1. D) Age below or above 65. E) CA 19-9 below or above the median. F) Treatment with chemotherapy or best supportive care.


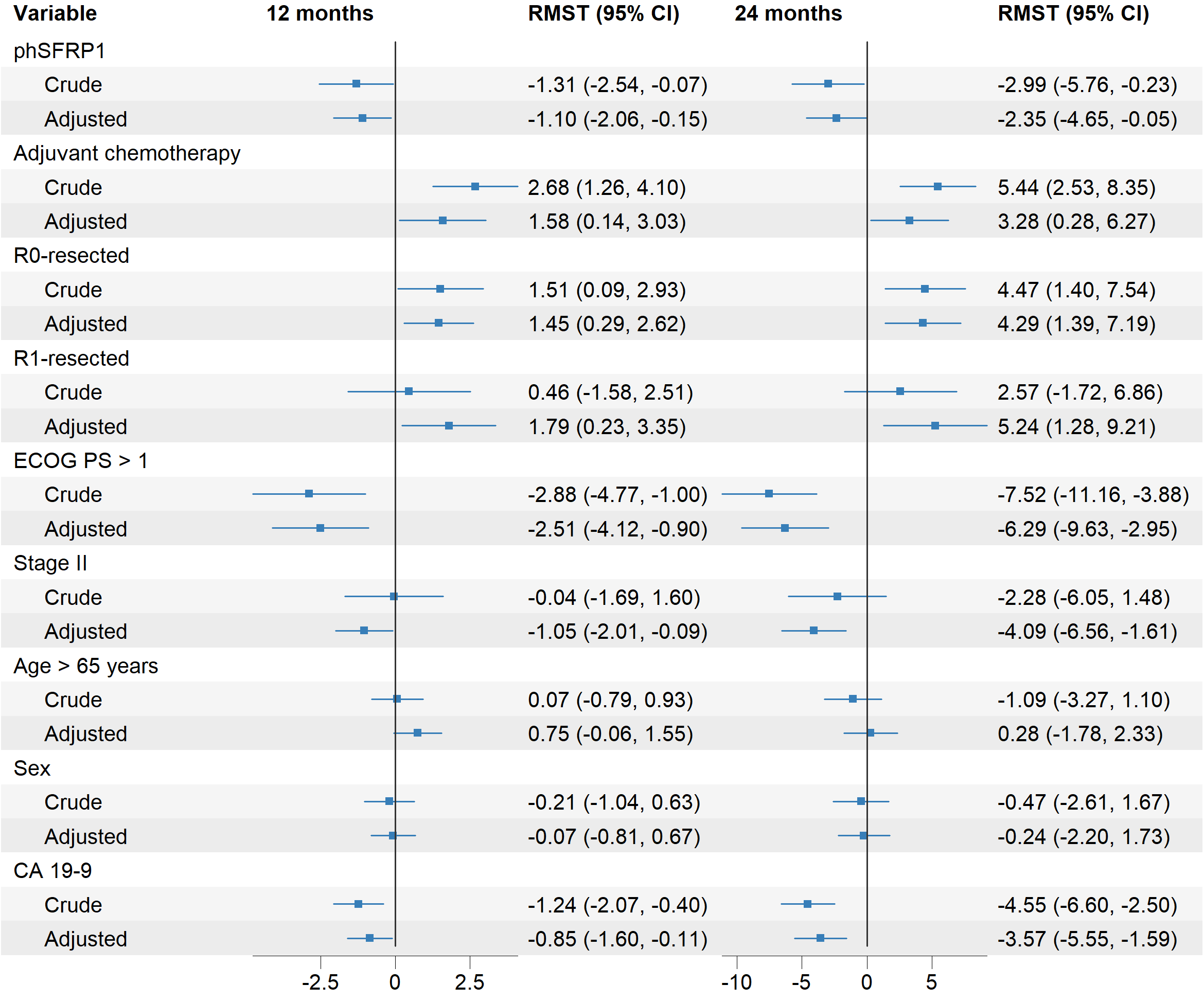


**Supplementary Figure 2.** Crude and adjusted differences in Restricted Mean Survival Time (RMST) in months between groups according to SFRP1 methylation status as well as the prognostic factors age > 65 years, PS, sex, treatment with adjuvant chemotherapy, stage of disease, CA 19-9 above the median, and resection status. Differences are calculated from baseline up to 12 and 24 months, respectively. Only cases with complete information on PS (n = 183). Increased survival is indicated by positive number in months, decreased survival is indicated by negative numbers.
